# Socioeconomic position, energy labelling and portion size selection: An online study comparing calorie and physical activity calorie equivalent (PACE) labelling in UK adults

**DOI:** 10.1016/j.appet.2021.105437

**Published:** 2021-11-01

**Authors:** Lucile Marty, Caterina Franzon, Andrew Jones, Eric Robinson

**Affiliations:** a Department of Psychological Sciences, University of Liverpool, Liverpool, UK; b Centre des Sciences Du Goût et de l’Alimentation, Agrosup Dijon, CNRS, INRAE, Université Bourgogne Franche-Comté, Dijon, France

**Keywords:** Energy labelling, PACE labelling, Portion size, SEP, SEP (socioeconomic position), BMI (body mass index)

## Abstract

Limited research has examined the impact of energy labelling on portion size selection. It is also unclear whether physical activity calorie equivalent (PACE) is more effective than standard kilocalorie (kcal) energy labelling in promoting healthier dietary behaviour and whether effectiveness varies based on socioeconomic position (SEP). In the present online study, 1667 UK adults of lower and higher SEP made virtual portion size selections for 18 common main meal foods under one of four conditions: kcal labelling only, PACE labelling only, kcal and PACE labelling, no labelling. Contrary to predictions, participants in the kcal labelling condition (+55 kcal, *p* < 0.001) chose larger portion sizes compared to the no labelling condition, whereas the PACE labelling (−17 kcal, *p* = 0.065) and no labelling condition did not significantly differ. The presence of PACE information on labels was associated with selection of significantly smaller portions when compared to labels that only included kcal information. Effects of labels on portion size selection were not moderated by participant SEP in primary analyses. The present study of virtual portion size selections suggests that kcal labelling resulted in larger portion size selections than no labelling, but this counter-intuitive effect was attenuated when kcal and PACE labelling were combined. Further research examining the impact of PACE labelling on real-world food selection in participants of lower and higher SEP is now warranted.

## 1 Introduction

The amount of food served at a meal (portion size) has been shown to have a causal effect on energy intake in both laboratory and real-world conditions (French et al., 2014; Haynes, Hardman, Halford, Jebb, & Robinson, 2020; Rolls, Morris, & Roe, 2002). There is evidence that people eat more when meal portion sizes are larger and this results in increased daily energy intake (Rolls, Roe, & Meengs, 2007). Likewise, recent evidence suggests that reducing portion sizes at main meals decreases daily energy intake (Haynes et al., 2020, Haynes et al., 2020). Reducing portion sizes has therefore been identified as a target for public health interventions to decrease energy intake and reduce obesity (Marteau, Hollands, Shemilt, & Jebb, 2015). Although in some contexts meal portion size is determined by others, self-selection of meal portion size is common (e.g. preparing food at home, selecting food at buffets). A number of factors have been associated with self-selected portion size. For example, being male, having overweight/obesity (as opposed to normal weight) and lower dietary restraint have all been found to be associated with choosing larger self-selected portion sizes in some studies (Labbe, Rytz, Brunstrom, Forde, & Martin, 2017; Lewis et al., 2015). However, there has been less research evaluating interventions to reduce self-selected portion sizes.

A common nutrition intervention approach is to provide information about the healthiness or energy content of meals or products through food labelling to encourage healthier food choices (Shangguan et al., 2019). Energy labelling of food sold outside of the home has recently been introduced through legislation in the US and is being considered in the UK (Cleveland, Simon, & Block, 2018; Robinson, Burton, Gough, Jones, & Haynes, 2019). Although evidence is mixed, some reviews have concluded that energy labelling results in consumers choosing lower energy menu options (Bleich et al., 2017; Crockett et al., 2018; Littlewood, Lourenço, Iversen, & Hansen, 2016). Yet, there is very limited evidence on the impact that energy labelling has on self-selected portion size. Two laboratory studies examined ad-libitum meal energy intake (as opposed to self-serving of portion size) and found no effect of the presence of energy labelling (Carbonneau et al., 2015; McCann et al., 2013). The most relevant direct evidence is from a study using a mock food buffet and energy labelling did not impact on self-selected portion size in this study (Brown, De Vlieger, Collins, & Bucher, 2017).

A different type of energy labelling that may be more effective is physical activity calorie equivalent (PACE) labelling. PACE labelling is thought to be easier to interpret in a meaningful way and therefore may be more likely to affect consumer behaviour (Daley, McGee, Bayliss, Coombe, & Parretti, 2020; Mason, Farley & Pallan, 2018; Bleich et al., 2012). A recent systematic review (Daley et al., 2020) of a range of study types concluded that PACE labelling may reduce energy intake, but the authors also found PACE information to be no more effective than standard nutrition information only, as did another review (Seyedhamzeh, Bagheri, Keshtkar, Qorbani, & Viera, 2018). However, a limitation of both reviews was that relatively few studies have directly compared PACE to standard energy labelling.

Due to the lack of evidence on the impact that energy labelling has on portion size selection, in the present online study we examined hypothetical portion size selection for a range of main meal foods in the presence vs. absence of energy labelling and tested the effects of both standard energy labelling (kcal) and PACE labelling. We hypothesised that both types of labelling would result in smaller self-selected portion sizes compared to no labelling and that PACE labelling would exert a larger effect than standard energy labelling.

A recent review concluded that there is some evidence that energy labelling may largely benefit people of higher, as opposed to lower SEP, and therefore widen health inequalities (Sarink et al., 2016). For an intervention like energy labelling to change behaviour, individuals are reliant on ‘executive function’; people need to attend to information (e.g., number of calories), hold the information in mind and then consciously act (Hall & Marteau, 2014). Likewise, for energy labelling to be effective, people presumably need to be motivated by health when making portion size selections (Grunert, Fernández-Celemín, Wills, genannt Bonsmann, & Nureeva, 2010). Because lower SEP is associated with reduced executive function and being less motivated by health when making food choices (Kao, Nayak, Doan, & Tarullo, 2018; Pechey, Monsivais, Ng, & Marteau, 2015), energy labelling may have a smaller effect on portion size selections in people of lower vs. higher SEP. However, some recent studies have not supported this hypothesis (Marty, Cook, Piernas, Jebb, & Robinson, 2020, Marty, Jones, & Robinson, 2020, Marty, Reed, Jones, & Robinson, 2021, Robinson, Smith, & Jones, 2021). In the present study, we therefore examined whether any effects of energy labelling (kcal or PACE) on portion size selection were moderated by SEP and explored whether SEP differences in executive function or food choice motives explained any differential effects of energy labelling on portion size selection by SEP. We hypothesised that effects of energy labelling may be smaller in lower SEP participants compared to higher SEP participants and this may be explained by SEP differences in executive functioning and health motives.

## 2 Methods

### 2.1 Data collection

Participants were recruited through the platform Prolific Academic (Peer, Brandimarte, Samat, & Acquisti, 2017) in April–May 2020. Participants were eligible to participate if they were UK residents, aged of 18 or above, fluent in English, had access to a computer with an internet connection, and had no dietary restrictions. Participants were asked to complete the study using their laptop or computer only (i.e. not on a mobile phone or tablet device). We intended to recruit a sample stratified by gender (approx. 50% male, 50% female) and highest educational qualification (approx. 50% A-level or below, 50% above A-level). Eligible participants who completed the study received monetary compensation in return for their participation (£2.09). The study was approved by the Health and Life Sciences Research Ethics Committee at the University of Liverpool (reference: 4612). All participants were informed that the purpose of the study was to understand eating behaviours and they provided their consent to participate.

### 2.2 Study design

This study was a randomised, controlled, pre-registered (see: https://osf.io/k369t/) online experiment developed using Inquisit Web version5.0.11 (Millisecond, Seattle, MA). Participants were randomly allocated to one of the four following conditions before performing a portion selection task: kcal labelling (kcal+/PACE-), PACE labelling as minutes to walk to burn off calories in food (kcal-/PACE+), kcal and PACE labelling (kcal+/PACE+), or no labelling (kcal-/PACE-). Randomisation with a 1:1:1:1 ratio across conditions was used.

### 2.3 Portion selection task

#### 2.3.1 Procedure

Participants were asked to choose the amount of food they would like to eat in 18 hypothetical meals (main dish only) sequentially displayed on the screen (for complete instructions see Additional file – section 1). The first picture of each dish showed 20 kcal of the dish. Participants were able to virtually serve themselves the amount of food they would like to eat by increasing or reducing the quantity on the plate using the arrow keys on their keyboard (right key to increase, left key to decrease). Energy content increased in 20 kcal intervals (min = 20 kcal, max = 1000 kcal). Participants had to increase the portion size at least once before confirming their choice using the spacebar. The energy content of the selected portion was recorded. The 18 dishes were presented in randomised order. The food pictures were of ready meals sold at supermarkets and have been used in previous research (Whitelock, Higgs, Brunstrom, Halford, & Robinson, 2018). For each of 18 dishes, 50 pictures reflected the increase in portion size. The dishes were common UK main meals (e.g., fish, chips and peas, spinach & ricotta tortellini with tomato sauce). See Additional file – section 2 for a full description.

#### 2.3.2 Interventions

**Fig. 1:**
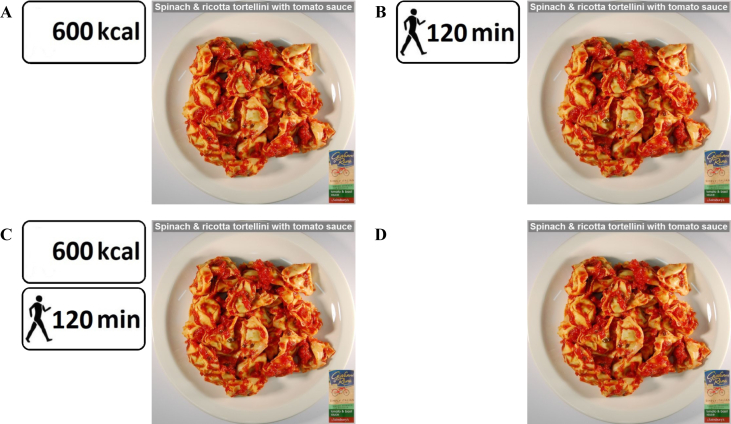
Example of a food picture in the portion selection task in the four experimental conditions, A: kcal+/PACE-, B: kcal-/PACE+, C: kcal+/PACE+, D: kcal-/PACE-.

Labelling was presented next to images of food portion sizes and were adapted from the labels used in Swartz et al. (Swartz, Dowray, Braxton, Mihas, & Viera, 2013). See Fig. 1. The kcal labels displayed the number of kcal in the dish and the PACE labels displayed the number of minutes of walking required to ‘burn off’ the amount of energy in the dish.^1^ The participants allocated to the kcal labelling condition (kcal+/PACE-) saw the kcal labels only and recommendations for daily energy intake: “On average women need 2,000 kcal per day and men need 2,500 kcal per day”. Participants allocated to the PACE labelling condition (kcal-/PACE+) saw the PACE labels only and recommendations for weekly physical activity as “According to physical activity recommendations, adults should aim to take part in at least 150 min of moderate intensity physical activity per week (brisk walk, swim, cycle)”. Participants allocated to the kcal and PACE labelling condition (kcal+/PACE+) saw both the kcal and PACE labels and recommendations on energy intake and physical activity. The participants in the no labelling condition (kcal-/PACE-) saw no labels and no recommendations. The values presented in the labels (kcal or PACE) next to the food pictures changed in response to increasing or decreasing the portion size.

### 2.4 Familiarity and liking

Participants were presented with the pictures of the 600 kcal portion of each of the 18 dishes, as UK public health guideline recommend energy consumption ≤600 kcal for a main meal and were asked: “Have you ever eaten this food?” with yes or no answers to measure familiarity, and “How much do you like this food?” with answers on a 100-point visual analogue scale (anchors: not at all, extremely) to measure liking.

### 2.5 Measures of SEP

We chose the primary measure of SEP to be education level because higher education level is associated with greater use of nutritional labels (Breck, Cantor, Martinez, & Elbel, 2014; Cowburn & Stockley, 2005). We collected two distinct measures of education level: highest educational qualification and total years in higher education (Galobardes et al., 2006). A-level or below qualifications were categorised as ‘lower education level’ whereas qualifications above A-level were categorised as ‘higher education level’. To account for both the level of qualification achieved and time spent in education, we calculated a continuous composite score (‘level of education’) as the mean of the z-scores for highest educational level and years in higher education. Participants were also asked to report the annual after-tax income of their household. Equivalised household income was calculated to adjust household income for household size and composition (Office for National Statistics, 2015). To measure perceived SEP participants rated where they believed they stood in society from 1 (worst off) to 10 (best off), using the MacArthur scale (Adler, Epel, Castellazzo, & Ickovics, 2000) of subjective social status (SSS).

### 2.6 Additional demographic measures

Age, gender, ethnicity, employment status, height, weight, dieting status and hunger state were recorded. Self-reported body mass index (BMI) was calculated in kg/m^2^. As participants were recruited during the social lockdown period in the UK due to the COVID-19 pandemic (2020), participants were asked if they suspected having or having had COVID-19 and how worried they were about their health.

### 2.7 Individual difference measures

#### 2.7.1 Inhibition (executive function measure)

Inhibition is the ability to suppress impulsive or automatic responses. A Stroop task was used to measure inhibition because performance on this task has been previously related to poverty and excess energy intake (Mani, Mullainathan, Shafir, & Zhao, 2013; Yang, Shields, Guo, & Liu, 2018). We implemented the classic Stroop task with keyboard responses. Participants saw names of colours presented in varying colour and were asked to indicate the colour of the word by key press as fast as they could without making too many errors. The task included congruent trials where the word and the colour it was presented in were the same (e.g. the word ‘blue’ presented in blue text), incongruent trials where colour word and the colour it was presented in were not the same (e.g. the word ‘blue’ presented in red text), and control trials with coloured rectangles in a mixed design. The task included four colours (red, green, blue, black), three colour-stimuli congruency conditions (congruent, incongruent and control), and 7 repetitions for a total of 84 trials (28 congruent, incongruent and control trials). We calculated the median reaction times (RTs) for correct responses in incongruent and congruent trials (Rousselet & Wilcox, 2018). The Stroop interference effect was calculated as the difference between the median RTs of the incongruent trials and the congruent trials [incongruent RT – congruent RT] for correct trials only. A larger interference score is indicative of poorer inhibition. We also calculated the proportion of correct responses in incongruent trials as a secondary outcome.

#### 2.7.2 Working memory (executive function measure)

Working memory is the ability to monitor the relevance of incoming stimuli and update information in memory in order to act in a goal-directed manner (e.g., sticking to healthy eating goals). We implemented a backwards digit-span task (Woods et al., 2011) on Inquisit because this task has been previously used to investigate executive functioning performance in individuals with excess energy intake (Yang et al., 2018). The task required participants to repeat series of digits (presented visually on screen) of increasing length in reversed order, via key presses. If participants made a correct response the subsequent trial moved up a level (addition of a digit), if the participants made an incorrect response the subsequent trial moved down a level (removal of a digit). The first trial was a sequence of two digits and the task consisted of 14 trials. Our primary outcome was the two-error maximum length as the last digit-span a participant got correct before making two consecutive errors and the maximum length as the maximal backward digit span that a participant recalled correctly during all 14 trials, as in (Marty, Jones, et al., 2020).

#### 2.7.3 Health and weight control motives

To assess participants' health and weight control motives in their food choices, we used the health subscale (6 items – Cronbach's α = 0.85; e.g., It is important to me that the food I eat on a typical day keeps me healthy) and weight control subscale (3 items – Cronbach's α = 0.80; e.g., It is important to me that the food I eat on a typical day is low in calories) from the Food Choice Questionnaire developed Steptoe et al. (Steptoe, Pollard, & Wardle, 1995) with responses ranging from 1 = Not at all important to 4 = Very important.

#### 2.7.4 Physical activity level

We used the International Physical Activity Questionnaire Short Version (IPAQ-SF) to assess physical activity levels (Craig et al., 2003). Participants were asked to record the number of times on a usual week and typical duration of activities varying in level of physical activity (e.g. vigorous-intensity activities, moderate-intensity activities, walking). Physical activity levels were estimated as MET (metabolic equivalent of task) minutes per week calculated for each type of activity by multiplying the self-reported duration by the number of times each type of activity was performed on a usual week by the MET score for each type of activity (walking: 3.3, moderate intensity: 4.0, vigorous intensity: 8.0 (Craig et al., 2003)). In line with scoring guidelines IPAQ Research Committee. (2005) activities below 10 min were recoded as 0 and activities over 180 min were recoded to be equal to 180 min.

### 2.8 Procedure

Participants were redirected from Prolific to Inquisit and randomised to one of the four experimental conditions. Participants first completed demographic questions including measures of SEP and completed the portion selection task and the executive functioning measures. Participants then completed the food choice motives items, IPAQ and rated their familiarity and liking of the dishes from the portion selection task. Finally, participants completed a questionnaire including items on the influence of the labelling interventions on their portion size selections and what they thought the aims of the study were (free text). The study had to be completed in one sitting.

### 2.9 Statistical analyses and sample size

We followed a pre-registered analysis protocol (https://osf.io/k369t/). Only participants who fully completed the study were included in analyses. We planned to exclude participants who failed at least one (of three) attention check (e.g., ‘How many times have you visited the planet Mars?‘) from analyses. The primary aim of the statistical analyses was to test the effect that the labelling interventions and SEP had on self-served energy. A linear mixed model was run in order to test the effect of labelling (four levels: kcal+/PACE-, kcal-/PACE+, kcal+/PACE+, kcal-/PACE-), highest educational qualification (two levels: higher, lower) and labelling*highest educational qualification on self-served energy, with participants and dishes set as random effects to account for correlation between repeated measures by the same participant and across food. To follow up any main effects of intervention condition, we planned to compare the four arms of the labelling intervention applying a Bonferroni correction (6 pairwise comparisons: p-value significance threshold = 0.05/6 = 0.008). Two researchers coded participants' free-text responses to identify aim guessing and coding discrepancies were resolved by a third researcher. We conducted sensitivity analyses to examine whether the pattern of results from the main analyses differed when 1/ excluding participants who identified the aims of the study (n = 115), 2/ substituting the categorical variable highest educational qualification by level of education (composite score) in the primary model, 3/ including hunger and liking as covariates in the primary model, and 4/ excluding any dish that was familiar to less than 50% of participants (one dish was excluded) or that was scored < 50 in liking on average (two dishes were excluded). As secondary analyses, the primary analysis was replicated using two alternative measures of SEP (equivalised income and subjective social status). As exploratory analyses, we examined whether the measures of inhibition, working memory, healthiness and weight control motives moderated the effect of the labelling intervention on self-served energy.^2^ All statistical analyses were performed using SAS version 9.3 (SAS Institute, Inc., 2012 SAS® 9.3. Cary, NC). Statistical tests level of significance was set at *p* < 0.05 for main and sensitivity analyses, p < 0.01 in secondary analyses, and *p* < 0.001 in exploratory analyses to account for multiple testing, unless otherwise specified.

*Sample size calculation.* A meta-analysis of six studies investigating the impact of kcal labelling on energy consumed from a single food did not demonstrate a statistically significant effect (Crockett et al., 2018). A meta-analysis of two studies of the effect of PACE labelling on energy consumed from foods/drinks showed a significant reduction of 14.4% (Daley et al., 2020). Due to the small number of studies included in this meta-analysis and therefore uncertainty in the size of effect PACE labelling may produce, we conservatively powered our study to be able to detect a smaller reduction in portion size between groups (kcal, PACE or kcal and PACE). An analytic sample size of 1,600 participants was feasible and allowed us to detect an 8% energy reduction between groups (kcal, PACE, kcal and PACE or no label) at power = .80 with α = 0.05. This sample size also provided sufficient power to detect a small interaction effect (f = 0.07, power = .80) between the labelling condition and SEP (Browne, Lahi, & Parker, 2009).

## 3 Results

### 3.1 Participants

**Fig. 2:**
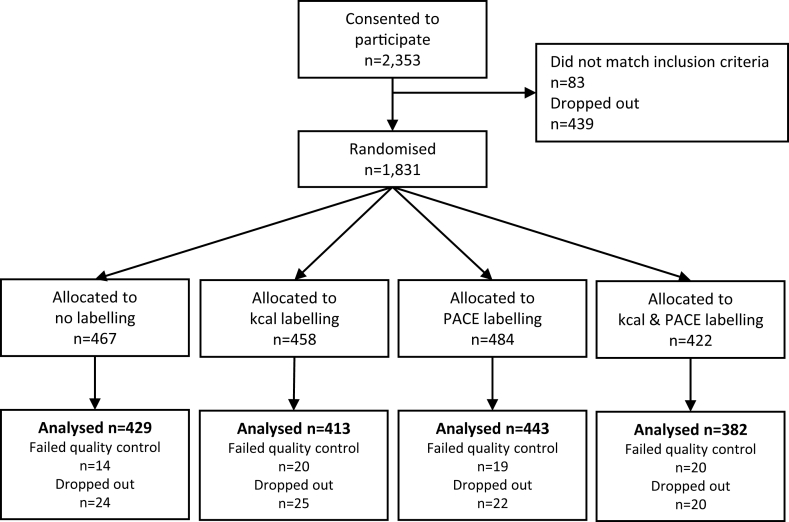
Survey flow chart ‘*Failed quality control’ indicates participants failing one or more attention checks*.

A total of 2353 participants consented to participate. Data from 1,667 (71%) who completed the study were analysed (Fig. 2). Sample characteristics overall and for participants of lower (≤A-level) and higher (>A-level) education are presented Table 1.

**Table 1:** Participant's characteristics overall and for participants of lower (A level or below) and higher (above A level) education.

	All (n = 1,667)	Lower education (n = 810)	Higher education (n = 857)	*p*-value^a^
**Age, years, mean (SD)**	36.9 (12.5)	37.3 (12.9)	36.6 (12.0)	0.230
**Gender, n (%),***female*	838 (50.3)	400 (49.4)	438 (51.1)	0.481
**Ethnicity, n (%),***white*	1511 (90.6)	774 (95.6)	737 (86.0)	<0.001
**Employment status, n (%),***full or part-time*	1251 (75.0)	563 (69.5)	688 (80.3)	<0.001
**Years of higher education, mean (SD)**	3.8 (2.4)	2.1 (1.3)	5.5 (2.1)	<0.001
**Equivalised household income, £, mean (SD)**	22952 (20680)	19695 (17748)	26029 (22696)	<0.001
**Subjective social status, mean (SD)**	5.2 (1.6)	4.8 (1.6)	5.6 (1.6)	<0.001
**BMI, kg/m**^**2**^**, mean (SD)**	26.8 (6.3)	27.3 (6.6)	26.3 (5.9)	0.002
*Missing or implausible*^b^*, n (%)*	14 (0.8)	11 (1.4)	3 (0.4)	
**Dieting status, n (%),***yes*	198 (11.9)	104 (12.8)	94 (11.0)	0.238
**Physical activity level, MET-minutes/week**^c^**, mean (SD)**				
*Total*	2362 (2186)	2359 (2405)	2365 (1957)	0.956
*Vigorous-intensity activity*	941 (1316)	935 (1469)	947 (1154)	0.856
*Moderate-intensity activity*	495 (740)	496 (789)	493 (691)	0.916
*Walking*	927 (910)	928 (955)	926 (867)	0.964

a T-tests comparing higher vs lower education groups for continuous variables, Chi-square tests for categorical variables.

b Excluding weight <30 kg or >250 kg, height <1.45 m or >3 m.

c Walking, Moderate and Vigorous time variables below ‘10 min’ were recoded to be equal to 0 and variables above ‘180 min’ were recoded to be equal to 180.

### 3.2 Familiarity and liking

On average, 83.9 ± 16.0% of the dishes were familiar to the participants (i.e., have been tried before) and the average liking of the dishes was 61.7 ± 11.9 on a scale from 0 to 100. The percentage of the dishes that were familiar to participants of lower education (82.1 ± 16.4%) was significantly lower than the percentage of the dishes that were familiar to participants of higher education (85.7 ± 15.5%, *t(1665)* = 4.61, *p* < 0.001). The average liking of the dishes for participants of lower education (61.3 ± 12.3) was not significantly different than the average liking of the dishes for participants of higher education (62.0 ± 11.6, *t(1665)* = 1.21, *p* = 0.226).

### 3.3 Effect of the labelling intervention and SEP on self-served energy

**Fig. 3:**
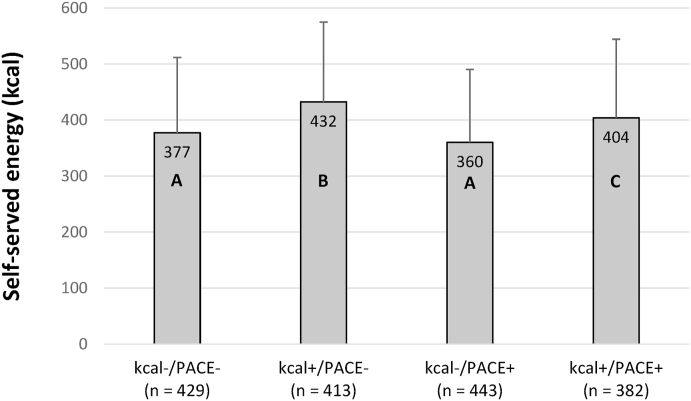
Average self-served energy by experimental condition (mean + SD). Least square means post-hoc comparisons: values with the same letter are not significantly different at Bonferroni corrected alpha level of 0.008.

Self-served energy across groups and experimental conditions is described Table 2. Self-served energy for each dish is reported in Additional file – section 3. In the linear mixed model there was a significant main effect of the labelling intervention (*F*(3,28^e+3^) = 21.96, *p* < 0.001), but no main effect of highest educational qualification (*F*(1,28^e+3^) = 3.33, *p* = 0.068) and no interaction effect (*F*(3,28^e+3^) = 1.59, *p* = 0.189) (see Additional file – section 4 for a complete description of the model). As the interaction between labelling and the highest educational qualification of the participants was not significant (*p* = 0.189), we next conducted pairwise comparisons between the four arms of the labelling intervention (Fig. 3). In the kcal+/PACE- (+55 kcal, 95% CI + 36 to +72, *p* < 0.001) and in the kcal+/PACE+ (+27 kcal, 95% CI + 8 to +45, *p* = 0.006) conditions, the participants served themselves statistically more energy than in the kcal-/PACE- condition. No difference was found in self-served energy between the kcal-/PACE+ and the kcal-/PACE- condition (−17 kcal, 95% CI -35 to 1, *p* = 0.065). In the kcal+/PACE+ condition participants served themselves more energy than in the kcal-/PACE+ condition (+44 kcal, 95% CI + 25 to +62, *p* < 0.001), but less than in the kcal+/PACE- condition (−28 kcal, 95% CI -47 to −9, *p* < 0.001). The amount of self-served energy was higher in the kcal+/PACE- condition than in kcal-/PACE+ condition (+72 kcal, 95% CI + 53 to +89, *p* < 0.001). The significant effect of labelling was replicated in all sensitivity analyses models (excluding aim guessers, using the composite score ‘level of education’, including hunger and liking as covariates, and excluding unfamiliar and disliked dishes) and in secondary analyses substituting highest educational qualification by equivalised income or subjective social status (see Additional file – sections 5, 5 and 6 for a complete description of the models). As the interaction between the labelling intervention and highest educational qualification was close to conventional significance in the sensitivity model excluding aim guessers (*p* = 0.067), we conducted exploratory analyses by comparing labelling conditions in lower and higher education participants separately (see Additional file – Table S6) and used a Bonferroni correction (*p* = 0.05/12 = 0.004). Self-served energy was significantly lower in the kcal-/PACE+ condition than in the kcal-/PACE- condition for participants of lower education (−40 kcal, *p* = 0.003), but not for participants of higher education (+4 kcal, *p* = 0.762). In line with the results of the main analysis, the presence of kcal labelling (kcal+) tended to be associated with greater self-served energy across higher and lower SEP participants.

**Table 2:** Self-served energy by group and experimental condition (mean ± SD).

	Lower education	Higher education
kcal-/PACE-	381 ± 132 (n = 209)	374 ± 137 (n = 220)
kcal+/PACE-	418 ± 137 (n = 194)	445 ± 146 (n = 219)
kcal-/PACE+	347 ± 134 (n = 224)	373 ± 125 (n = 219)
kcal+/PACE+	402 ± 130 (n = 183)	405 ± 150 (n = 199)

### 3.4 Moderators of the labelling intervention

None of the measures of inhibition, working memory, health and weight control motives moderated the effect of the labelling intervention on self-served energy in analyses. See Additional file – section 7.

### 3.5 Portion selection task questionnaire

Participants reported that the portion size they selected was influenced by how many calories they thought were in the dishes to a larger extent in all the labelling conditions compared to the no labelling condition. Participants also reported that the portion size they selected was influenced by how much physical activity they thought they would have to do to burn off the calories that were in the dishes to a larger extent in the PACE labelling conditions compared to the no labelling condition. See Additional file – section 8 for results in full.

## 4 Discussion

In the present online study examining virtual main meal portion size selection, contrary to predictions, the presence of energy labelling in kcal resulted in participants selecting larger portion sizes compared to when no labelling was present. Participants exposed to kcal and PACE labelling combined selected smaller portion sizes than participants exposed to kcal labelling only, but portion sizes were still significantly larger in the kcal and PACE labelling condition than when no labelling was present. Participants in the PACE only labelling condition served themselves similar portions as participants in the no labelling condition. We did not find evidence that the effect of labelling condition on portion size selection was moderated by SEP in primary analyses.

The tendency for labelling including kcal information to increase selected portion sizes was unexpected, as research to date has suggested that energy labelling is associated with small reductions to amount of energy ordered (Bleich et al., 2017; Crockett et al., 2018; Littlewood et al., 2016) or no impact on self-selected portion size. (Carbonneau et al., 2015; McCann et al., 2013; Brown et al., 2017). Although participants selected portions of common main meal foods that they reported liking and being familiar with, portion size selections in the no labelling control condition were relatively low in energy (377 ± 134 kcal). We therefore speculate that under these conditions the presence of kcal labelling may have resulted in participants feeling that they could serve themselves more due to the discrepancy between the amount of energy of their portion and daily energy requirements displayed alongside kcal labels; a form of ‘backfire effect’. In a similar vein, a recent study found that energy labelling increased energy consumption when participants expected a food to be higher in energy than labelling indicated (Tangari, Bui, Haws, & Liu, 2018) and the present findings may also be in part explained by this process. PACE information alone did not result in participants serving significantly smaller portions when compared to the no labelling condition. Participants selected smaller portions in the kcal and PACE labelling vs. kcal labelling only condition. Therefore, although PACE labelling did not reduce portion size selection relative to no labelling, findings are consistent with recent suggestions the inclusion of PACE information about food (alongside kcal labelling) may be beneficial (Daley et al., 2020).

### 4.1 Effect of labelling across SEP

There was no evidence of effects of labelling being moderated by participant SEP in our primary analyses. In a planned analysis excluding participants that guessed the aims of the study we found some weak evidence that participants in the PACE labelling condition chose smaller portions than those in the no labelling condition among participants of lower, but not higher SEP. PACE labelling only was associated with smaller portions compared to kcal labelling only in both participants of higher and lower SEP, whereas kcal and PACE labelling combined did not differ to the no labelling condition in both participants of higher and lower SEP. However, findings concerning SEP should be interpreted with caution as they relate to exploratory follow-up analyses of a non-significant interaction effect (p = 0.067) from a planned sensitivity analysis. If these findings are replicated it will be of importance to consider why PACE labelling may be effective in reducing portion size selections in lower but not higher SEP participants. Previous research that has suggested that energy labelling may have a larger effect among higher SEP groups (Sarink et al., 2016) and one line of argument is that information provision based health interventions (such as food labelling) may be ineffective among lower SEP populations (Adams, Mytton, White, & Monsivais, 2016). However, the present findings may suggest that PACE labelling could be beneficial for lower SEP populations when making portion size selections. It may be that because nutrition knowledge and health literacy tends to increase with SEP (Parmenter, Waller, & Wardle, 2000; Stormacq, Van den Broucke, & Wosinski, 2018), the relatively easy to understand nature of PACE labelling may motivate health conscious lower SEP participants who find it difficult to understand kcal information. Given the exploratory nature of our findings relating to SEP and PACE labelling, further research examining SEP responses to energy in kcal vs. PACE labelling will now be valuable.

### 4.2 Strengths and limitations

There are strengths and limitations to the present study. Analyses were pre-registered and we were able to sample an SEP diverse and large group of UK adults. In addition, portion size selection was made for a wide range of common main meal foods in the UK that have been used in previous portion size selection research (Brunstrom, Shakeshaft, & Scott-Samuel, 2008; Whitelock et al., 2019). Labelling (e.g. kcal content, PACE) was displayed on screen next to the food and values increased/decreased in response to participants altering the amount of food displayed on the plate, which is not representative of how information would typically be presented in the real-world. Participants made hypothetical portion size selections from 2D photographs of food and although previous research has shown that hypothetical measures like this serve as a reasonable proxy to food consumption (Robinson, te Raa, & Hardman, 2015; Wilkinson et al., 2012), we do not know if the same results would be observed for actual food consumption. Portion size selections tended to be relatively low in energy content across all conditions. It would therefore now be valuable to examine whether similar findings would be observed for very high energy foods or other food type dimensions (Ikonen, Sotgiu, Aydinli, & Verlegh, 2020), as under such conditions kcal labelling may lead to differential effects on portion selection.

## 5 Conclusions

The present study of virtual portion size selections suggests that kcal labelling resulted in larger portion size selections than no labelling, but this counter-intuitive effect was attenuated when kcal and PACE labelling were combined. Further research examining the impact of PACE labelling on real-world food selection in participants of lower and higher SEP is now warranted.

## Availability of data and materials

The study dataset and registered protocol is available on the Open Science Framework repository at https://osf.io/k369t/.

## Funding

This project has received funding from the 10.13039/501100000781European Research Council (ERC) under the European Union's Horizon 2020 research and innovation programme (Grant reference: PIDS, 803194).

## Author contributions

All authors contributed to designing the research. CF and LM analysed the data. All authors contributed to drafting of the manuscript and approved the final manuscript.

## Ethical approval and consent to participate

Studies were approved by the University of Liverpool, Health and Life Sciences Research Ethics (reference: 4612).

## Consent for publication

N/A.

## Declaration of competing interest

No authors report conflicts of interest, ER has previously received research funding from the American Beverage Association and Unilever for projects unrelated to the present work.

## Footnotes

1 To be consistent with previous research, PACE information was based on an adult weighing 70 kg (4 min of moderate to vigorous walking being equivalent to 20 kcals).

2 As detailed in the results section, we did not find an interaction between labelling and highest educational level. Consequently, the moderated mediation analysis that was planned to investigate the potential mediators of the differential effect of labelling in lower vs. higher SEP participants was not performed (see pre-registered analysis plan: https://osf.io/k369t/).
