# Supplementary material for: Socioeconomic position, energy labelling and portion size selection: An online study comparing calorie and physical activity calorie equivalent (PACE) labelling in UK adults
Source: Appetite. 2021 Nov 1;166:105437. doi: 10.1016/j.appet.2021.105437 (PMC8385415; doi:10.1016/j.appet.2021.105437)
Supplement: Multimedia component 1 [file mmc1.docx]

**Additional file**

**Table of contents**

[1. Instructions for the portion selection task 2](#_Toc68537430)

[2. Description of the 18 dishes presented as food pictures to the participants 2](#_Toc68537431)

[3. Portion size selection for the 18 dishes 4](#_Toc68537432)

[4. Main analyses: effects of the intervention and highest educational qualification on self-served energy 5](#_Toc68537433)

[5. Sensitivity analyses 7](#_Toc68537434)

[6. Secondary analyses 12](#_Toc68537435)

[7. Moderators of the labelling intervention 13](#_Toc68537436)

[8. Portion selection task questionnaire 17](#_Toc68537437)

**Tables**

[**Table S1.** Description of the 18 dishes presented as food pictures to the participants 2](#_Toc68537728)

[**Table S2.** Portion size selection for each dish, mean (SD) 4](#_Toc68537729)

[**Table S3.** Fixed effect statistics of linear mixed model with participants and dishes as random, dependant variable: self-served energy (kcal), n = 1667 5](#_Toc68537730)

[**Table S4.** Fixed effect statistics of linear mixed models with participants and dishes as random, dependant variables: self-served energy (kcal) 7](#_Toc68537731)

[**Table S5.** Least square means (± standard error) by experimental condition, values with the same letter are not significantly different at alpha level of 0.008 10](#_Toc68537732)

[**Table S6.** Comparison of least square means (± standard error) by experimental condition and group of highest educational qualification (lower and higher) from Model 1 (excluding aim guessers) 11](#_Toc68537733)

[**Table S7.** Fixed effect statistics of linear mixed models with participants and dishes as random, dependant variables: self-served energy (n = 1667) 12](#_Toc68537734)

[**Table S8.** Inhibition (Stroop task), working memory (backward digit-span task), healthiness and weight control motives overall and in participants of lower and higher education 13](#_Toc68537735)

[**Table S9.** Fixed effect statistics of linear mixed models with participants and dishes as random, dependant variables: self-served energy 14](#_Toc68537736)

[**Table S10.** Description of the answers to the debriefing questionnaire overall and for participants of lower and higher education 17](#_Toc68537737)

[**Table S11.** ANOVA models, dependant variables: items 1 and 2 of the portion selection task questionnaire (n=1667) 19](#_Toc68537738)

1. **Instructions for the portion selection task**

The instructions the participants were shown before starting the portion selection task were as follows: “In this task, you will be shown pictures of 18 different dishes. For each dish, you will be asked to decide how much you would like to eat as if you were eating it as main meal. You can change the quantity using the arrow keys on your keyboard: tap on the right arrow to increase the quantity, on the left one to decrease the quantity, and use the space bar to select the portion size you would like to eat. Before you make your choice, take the time to explore the full range of portion sizes. You need to increase the amount of food at least once to be able to select a portion and continue.” For participants in kcal+ conditions: “For each portion, the label on the left will show you the calorie content.” For participants in PACE+ conditions: “For each portion, the label on the left will show you the physical activity calorie equivalent, as the number of minutes of walking needed to burn off the calories in the meal.”

1. **Description of the 18 dishes presented as food pictures to the participants**

For each of 18 dishes, 50 pictures reflected the increase in portion size. The number of calories increased 20 kcal by 20 kcal from a minimum of 20 kcal (first picture) to a maximum of 1000 kcal (50th picture). **Table S1** shows the energy density and the composition of the 30^th^ picture (i.e., 600 kcal) for each of the 18 dishes.

**Table S1.** Description of the 18 dishes presented as food pictures to the participants

|  | **Energy density**  (kcal/100g) | **30^th^ picture**  600 kcal^a^  (g) |
| --- | --- | --- |
| Beef in black beans sauce with rice  Beef  Rice | 130 | 261  201 |
| Beef stew with dumplings  Beef  Dumplings | 167 | 243  117 |
| Chicken and bacon pasta | 139 | 432 |
| Chicken, chips and beans  Chicken  Chips  Beans | 198 | 93  126  84 |
| Chicken korma with rice  Chicken  Rice | 154 | 213  177 |
| Chicken salad  Cucumber  Salad  Chicken  Croutons  Dressing  Parmesan | 98 | 219  168  132  42  36  15 |
| Couscous and salad  Couscous  Salad | 180 | 306  27 |
| Fish, chips and peas  Fish  Chips  Peas | 171 | 98  176  76 |
| Grilled white fish with tomato and bean salad  Fish  Bean salad | 76 | 327  465 |
| Ham and mushroom carbonara | 199 | 302 |
| Lasagne and peas  Lasagne  Peas | 137 | 357  81 |
| Macaroni cheese | 183 | 327 |
| Mushroom risotto | 130 | 462 |
| Peperoni pizza | 271 | 221 |
| Sausages, mashed potatoes and peas  Sausages  Potatoes  Peas | 114 | 141  291  93 |
| Spaghetti Bolognese | 141 | 426 |
| Spinach & Ricotta tortellini with tomato sauce | 317 | 189 |
| Vegetable biryani | 127 | 471 |

^a^ UK public health guideline recommend energy consumption ≤ 600 kcal for a main meal

1. **Portion size selection for the 18 dishes**

The mean energy (kcal) selected for all dishes is presented **Table S2**.

**Table S2.** Portion size selection, mean (SD)

|  | **Mean portion size**  **(kcal)** |
| --- | --- |
| Beef in black beans sauce with rice | 321 (163) |
| Beef stew with dumplings | 540 (258) |
| Chicken and bacon pasta | 444 (224) |
| Chicken, chips and beans | 626 (254) |
| Chicken korma with rice | 353 (165) |
| Chicken salad | 203 (117) |
| Couscous and salad | 180 (116) |
| Fish, chips and peas | 602 (211) |
| Grilled white fish with tomato and bean salad | 249 (153) |
| Ham and mushroom carbonara | 444 (248) |
| Lasagne and peas | 508 (227) |
| Macaroni cheese | 346 (192) |
| Mushroom risotto | 320 (202) |
| Peperoni pizza | 439 (226) |
| Sausages, mashed potatoes and peas | 411 (183) |
| Spaghetti Bolognese | 406 (178) |
| Spinach & Ricotta tortellini with tomato sauce | 363 (201) |
| Vegetable biryani | 308 (178) |

1. **Main analyses: effects of the intervention and highest educational qualification on self-served energy**

The primary model was a linear mixed model testing the effect of labelling (four levels: kcal+/PACE-, kcal-/PACE+, kcal+/PACE+, kcal-/PACE-), highest educational qualification (two levels: higher, lower) and labelling*highest educational qualification on self-served energy, with participants and dishes set as random effects to account for correlation between repeated measures by the same participant and across the food items (**Table S3**).

**Table S3.** Fixed effect statistics of linear mixed model with participants and dishes as random, dependant variable: self-served energy (kcal), n = 1667

|  | **Type III effects** | | ***Estimate*** | ***95% LCL*** | ***95% UCL*** |
| --- | --- | --- | --- | --- | --- |
| **Main model** | ***F*** | ***p*** |  |  |  |
| Intercept^a^  Intervention  Kcal labelling  PACE labelling  Kcal & PACE labelling  *No labelling*  Education  Higher  *Lower*  Intervention*education  Kcal labelling*higher  *Kcal labelling*lower*  PACE labelling*higher  *PACE labelling*lower*  Kcal & PACE labelling*higher  *Kcal & PACE labelling*lower*  *No labelling*higher*  *No labelling*lower* | 21.96  3.33  1.59 | < 0.001  0.068  0.189 | 380.75  37.23  -33.32  21.61  0  -6.66  0  33.55  0  32.46  0  9.53  0  0  0 | 315.50  10.56  -59.05  -5.48  .  -32.50  .  -3.39  .  -3.80  .  -28.14  .  .  . | 446.00  63.91  -7.59  48.70  .  19.19  .  70.48  .  68.72  .  47.20  .  .  . |

^a^ Reference group: no labelling, lower education; parameters estimates must be interpreted as difference with the reference group.

As an exploratory analysis, we also ran an adjusted model including age, gender, hunger, dieting status, BMI and liking as covariates. Findings produced a similar pattern of results as in the main analyses. There was a significant main effect of the labelling intervention (*F*(3,28e+3) = 23.12, *p* < 0.001), a main effect of highest educational qualification (*F*(1,28e+3) = 4.60, *p* = 0.032) and no interaction effect (*F*(3,28e+3) = 1.25, *p* =0.290).

1. **Sensitivity analyses**

We conducted sensitivity analyses to examine whether the pattern of results from the main analyses differed when 1/ Model 1: excluding the aim guessers (n=115), 2/ Model 2: substituting the categorical variable highest educational qualification by level of education (composite score) in the main model, 3/ Model 3: including hunger and liking as covariates in the main model, and 4/ Model 4: excluding any dish that was familiar to less than 50% of participants (one dish excluded) or that was scored < 50 in liking on average (two dishes were excluded) (**Table S4 and Table S5**). The results showed a significant main effect of education in models 1 and 2 with participants of higher education serving themselves less energy than participants of lower education. As opposed to the results for the main model, self-served energy in the kcal and PACE labelling condition was not different from self-served in the no labelling condition in models 1, 3 and 4.

**Table S4.** Fixed effect statistics of linear mixed models with participants and dishes as random, dependant variables: self-served energy (kcal)

|  | **Type III effects** | | ***Estimate*** | ***95% LCL*** | ***95% UCL*** |
| --- | --- | --- | --- | --- | --- |
|  | ***F*** | ***p*** |  |  |  |
| **Model 1 (n = 1552)**  Intercept^a^  Intervention  Kcal labelling  PACE labelling  Kcal & PACE labelling  *No labelling*  Education  Higher  *Lower*  Intervention*education  Kcal labelling*higher  *Kcal labelling*lower*  PACE labelling*higher  *PACE labelling*lower*  Kcal & PACE labelling*higher  *Kcal & PACE labelling*lower*  *No labelling*higher*  *No labelling*lower* | 19.34  3.89  2.39 | < 0.001  0.048  0.067 | 380.75  36.05  -39.60  21.24  0  -6.66  0  32.99  0  43.74  0  4.91  0  0  0 | 315.30  9.12  -65.88  -6.76  .  -32.54  .  -4.59  .  6.26  .  -33.91  .  .  . | 446.20  62.99  -13.33  49.24  .  19.22  .  70.58  .  81.22  .  43.73  .  .  . |
| **Model 2 (n = 1667)**  Intercept^a^  Intervention  Kcal labelling  PACE labelling  Kcal & PACE labelling  *No labelling*  Level of education  Intervention*level of education  Kcal labelling*level of education  PACE labelling*level of education  Kcal & PACE*level of education  *No labelling*level of education* | 22.35  4.39  2.33 | < 0.001  0.036  0.072 | 377.33  54.68  -16.88  26.50  0  -4.64  24.23  17.09  6.97  0 | 313.66  36.25  -35.00  7.69  .  -17.95  4.83  -1.95  -12.77  . | 441.00  73.11  1.23  45.30  .  8.68  43.62  36.12  26.70  . |
| **Model 3 (n = 1667)**  Intercept^a^  Intervention  Kcal labelling  PACE labelling  Kcal & PACE labelling  *No labelling*  Education  Higher  *Lower*  Intervention*education  Kcal labelling*higher  *Kcal labelling*lower*  PACE labelling*higher  *PACE labelling*lower*  Kcal & PACE labelling*higher  *Kcal & PACE labelling*lower*  *No labelling*higher*  *No labelling*lower*  Hunger  Liking | 23.00  2.88  1.56  10.31  7926.04 | < 0.001  0.090  0.196  0.001  < 0.001 | 203.46  36.66  -33.78  14.89  0  -9.57  0  30.05  0  34.04  0  16.61  0  0  0  0.38  2.69 | 143.53  11.77  -57.78  -10.38  .  -33.68  .  -4.41  .  0.22  .  -18.52  .  .  .  0.15  2.63 | 263.38  61.55  -9.78  40.16  .  14.54  .  64.51  .  67.86  .  51.74  .  .  .  0.61  2.74 |
| **Model 4 (n = 1667)**  Intercept^a^  Intervention  Kcal labelling  PACE labelling  Kcal & PACE labelling  *No labelling*  Education  Higher  *Lower*  Intervention*education  Kcal labelling*higher  *Kcal labelling*lower*  PACE labelling*higher  *PACE labelling*lower*  Kcal & PACE labelling*higher  *Kcal & PACE labelling*lower*  *No labelling*higher*  *No labelling*lower* | 20.30  1.79  1.85 | < 0.001  0.181  0.135 | 414.99  31.72  -40.82  17.71  0  -12.12  0  38.77  0  36.56  0  11.01  0  0  0 | 348.31  3.53  -68.01  -10.91  .  -39.43  .  -0.25  .  -1.75  .  -28.79  .  .  . | 481.66  59.90  -13.63  46.34  .  15.19  .  77.79  .  74.87  .  50.81  .  .  . |

^a^ Reference group: no labelling, lower education; parameters estimates must be interpreted as difference with the reference group.

**Table S5.** Least square means (± standard error) by experimental condition, values with the same letter are not significantly different at alpha level of 0.008

|  | **Main Model** | **Model 1** | **Model 2** | **Model 3** | **Model 4** |
| --- | --- | --- | --- | --- | --- |
| No labelling | 377 ± 30 **A** | 377 ± 30 **AB** | 377 ± 30 **A** | 379 ± 27 **AB** | 409 ± 30 **AB** |
| Kcal labelling | 431 ± 30 **B** | 430 ± 30 **C** | 432 ± 30 **B** | 430 ± 27 **C** | 460 ± 30 **C** |
| PACE labelling | 360 ± 30 **A** | 360 ± 30 **A** | 360 ± 30 **A** | 362 ± 27 **A** | 386 ± 30 **A** |
| Kcal & PACE | 404 ± 30 **C** | 401 ± 30 **B** | 404 ± 30 **C** | 402 ± 27 **B** | 432 ± 30 **B** |

As the interaction between the labelling intervention and highest educational qualification was marginally significant in Model 1 excluding aim guessers (*p* = 0.067), we conducted exploratory pairwise comparisons between the four arms of the labelling intervention in the groups of lower and higher education (**Table S6**). A Bonferroni correction with 12 comparisons was applied leading to a p-value significance threshold of 0.05/12 = 0.004. Self-served energy was significantly lower in the PACE labelling condition than in the no labelling condition for participants of lower education but not for participants of higher education. Self-served energy was significantly higher in the kcal labelling condition than in the no labelling condition for participants of higher education but not for participants of lower education.

**Table S6.** Comparison of least square means (± standard error) by experimental condition and group of highest educational qualification (lower and higher) from Model 1 (excluding aim guessers)

| **Lower education (n = 767)** |  | **No labelling** | **Kcal** | **PACE** | **Kcal & PACE** |
| --- | --- | --- | --- | --- | --- |
|  | Mean ± SE | 381 ±31 | 417 ± 31 | 341 ± 31 | 402 ± 31 |
| No labelling | 381 ±31 | - | *p* = 0.009 | *p* = 0.003 | *p* = 0.137 |
| Kcal labelling | 417 ± 31 |  | - | *p* < 0.001 | *p* = 0.311 |
| PACE labelling | 341 ± 31 |  |  | - | *p* < 0.001 |
| Kcal & PACE labelling | 402 ± 31 |  |  |  | - |
| **Higher education (n = 785)** |  | **No labelling** | **Kcal** | **PACE** | **Kcal & PACE** |
|  | Mean ± SE | 374 ± 31 | 443 ± 31 | 378 ± 31 | 400 ± 31 |
| No labelling | 374 ± 31 | - | *p* < 0.001 | *p* = 0.762 | *p* = 0.057 |
| Kcal labelling | 443 ± 31 |  | - | *p* < 0.001 | *p* = 0.002 |
| PACE labelling | 378 ± 31 |  |  | - | *p* = 0.124 |
| Kcal & PACE labelling | 400 ± 31 |  |  |  | - |

1. **Secondary analyses**

The main model was replicated using two alternative measures of socioeconomic position (equivalised income and subjective social status) and these analyses led to the same pattern of results as the main model (**Table S7**).

**Table S7.** Fixed effect statistics of linear mixed models with participants and dishes as random, dependant variables: self-served energy (n = 1667)

|  | **Type III effects** | | ***Estimate*** | ***95% LCL*** | ***95% UCL*** |
| --- | --- | --- | --- | --- | --- |
|  | ***F*** | ***p*** |  |  |  |
| **Equivalised income**  Intercept^a^  Intervention  Kcal labelling  PACE labelling  Kcal & PACE labelling  *No labelling*  Equivalised income  Intervention*equivalised income  Kcal labelling*equivalised income  PACE labelling*equivalised income  Kcal & PACE*equivalised income  *No labelling*equivalised income* | 10.01  0.70  0.08 | < 0.001  0.402  0.973 | 370.22  55.96  -18.22  29.87  0  0.00032  -0.00005  0.00002  -0.00016  0 | 304.08  24.79  -45.50  0.79  .  -0.00043  -0.00119  -0.00088  -0.00114  . | 436.37  87.14  9.07  58.94  .  0.00108  0.00109  0.00092  0.00081  . |
| **Subjective Social Status (SSS)**  Intercept^a^  Intervention  Kcal labelling  PACE labelling  Kcal & PACE labelling  *No labelling*  SSS  Intervention*SSS  Kcal labelling*SSS  PACE labelling*SSS  Kcal & PACE labelling*SSS  *No labelling*SSS* | 3.16  1.26  0.33 | 0.024  0.262  0.806 | 393.87  65.89  -30.81  11.11  0  -3.19  -2.00  2.65  2.98  0 | 316.68  3.84  -91.70  -50.44  .  -11.02  -13.31  -8.49  -8.24  . | 471.05  127.94  30.07  72.66  .  4.64  9.30  13.79  14.20  . |

^a^ Reference group: no labelling, lower education; parameters estimates must be interpreted as difference with the reference group.

1. **Moderators of the labelling intervention**

We compared the measures of inhibition, working memory, healthiness and weight control motives in participants of lower and higher education (**Table S8**). We then substituted highest educational qualification in the main model by these measures and there were no significant interactions with the labelling intervention at alpha level = 0.001 (**Table S9**).

**Table S8.** Inhibition (Stroop task), working memory (backward digit-span task), healthiness and weight control motives overall and in participants of lower and higher education

|  | **All**  **(n=1,667)** | **Lower education**  **(n=810)** | **Higher education**  **(n=857)** | ***p*-value^a^** |
| --- | --- | --- | --- | --- |
| **Inhibition**  Proportion of correct responses in incongruent trials  Interference Stroop effect (ms)  **Working memory**  Two-error maximum length  Maximum length  **Food choice motives**  Healthiness  Weight control | 0.90 ± 0.12  239 ± 233  5.9 ± 1.8  6.7 ± 1.7  2.7 ± 0.6  2.3 ± 0.7 | 0.90 ± 0.13  237 ± 240  5.8 ± 1.8  6.6 ± 1.7  2.6 ± 0.6  2.3 ± 0.7 | 0.91 ± 0.11  241 ± 226  6.1 ± 1.8  6.9 ± 1.6  2.8 ± 0.6  2.3 ± 0.7 | 0.002  0.722  0.001  0.001  < 0.001  0.603 |

**Table S9.** Fixed effect statistics of linear mixed models with participants and dishes as random, dependant variables: self-served energy

|  | **Type III effects** | | ***Estimate*** | ***95% LCL*** | ***95% UCL*** |
| --- | --- | --- | --- | --- | --- |
|  | ***F*** | ***p*** |  |  |  |
| **Moderator = Proportion of correct responses in incongruent trials**  Intercept^a^  Intervention  Kcal labelling  PACE labelling  Kcal & PACE labelling  *No labelling*  Moderator  Intervention*moderator  Kcal labelling*moderator  PACE labelling*moderator  Kcal & PACE labelling*moderator  *No labelling*moderator* | 5.26  23.52  4.50 | 0.001  < 0.001  0.004 | 270.90  -48.18  -108.26  150.39  0  117.40  112.50  102.07  -136.78  0 | 150.53  -200.27  -244.00  17.80  .  12.70  -53.51  -46.86  -282.11  . | 391.28  103.91  27.47  282.98  .  222.10  278.51  251.01  8.56  . |
| **Moderator = Stroop effect**  Intercept^a^  Intervention  Kcal labelling  PACE labelling  Kcal & PACE labelling  *No labelling*  Moderator  Intervention*moderator  Kcal labelling*moderator  PACE labelling*moderator  Kcal & PACE labelling*moderator  *No labelling*moderator* | 12.83  8.56  3.88 | < 0.001  0.003  0.009 | 386.11  68.71  -8.74  9.37  0  -0.04  -0.06  -0.04  0.07  0 | 320.94  42.05  -34.42  -17.00  .  -0.092  -0.14  -0.12  -0.01  . | 451.27  95.37  16.95  35.74  .  0.02  0.03  0.04  0.15  . |
| **Moderator =Two-error maximum length**  Intercept^a^  Intervention  Kcal labelling  PACE labelling  Kcal & PACE labelling  *No labelling*  Moderator  Intervention*moderator  Kcal labelling*moderator  PACE labelling*moderator  Kcal & PACE labelling*moderator  *No labelling*moderator* | 5.05  3.42  0.77 | 0.002  0.065  0.509 | 351.26  81.12  -35.14  41.61  0  4.36  -4.38  3.08  -2.50  0 | 275.05  21.36  -95.57  -22.53  .  -2.14  -14.04  -6.61  -12.82  . | 427.46  140.89  25.30  105.74  .  10.86  5.27  12.77  7.82  . |
| **Moderator = Maximum length**  Intercept^a^  Intervention  Kcal labelling  PACE labelling  Kcal & PACE labelling  *No labelling*  Moderator  Intervention*moderator  Kcal labelling*moderator  PACE labelling*moderator  Kcal & PACE labelling*moderator  *No labelling*moderator* | 2.81  0.11  0.54 | 0.038  0.739  0.652 | 368.35  62.52  -38.49  57.52  0  1.32  -1.11  3.18  -4.60  0 | 285.92  -11.78  -114.54  -21.51  .  -5.83  -11.86  -7.74  -15.96  . | 450.79  136.83  37.57  136.56  .  8.47  9.63  14.09  6.77  . |
| **Moderator = Healthiness**  Intercept^a^  Intervention  Kcal labelling  PACE labelling  Kcal & PACE labelling  *No labelling*  Moderator  Intervention*moderator  Kcal labelling*moderator  PACE labelling*moderator  Kcal & PACE labelling*moderator  *No labelling*moderator* | 2.67  12.31  0.51 | 0.046  < 0.001  0.676 | 420.06  69.18  -30.57  62.72  0  -15.55  -5.71  4.65  -13.21  0 | 329.02  -16.13  -114.28  -24.00  .  -37.54  -36.38  -25.37  -44.03  . | 511.11  154.48  53.15  149.45  .  6.45  24.95  34.68  17.61  . |
| **Moderator = Weight control**  Intercept^a^  Intervention  Kcal labelling  PACE labelling  Kcal & PACE labelling  *No labelling*  Moderator  Intervention*moderator  Kcal labelling*moderator  PACE labelling*moderator  Kcal & PACE labelling*moderator  *No labelling*moderator* | 2.54  47.32  0.48 | 0.055  < 0.001  0.699 | 444.27  48.97  -17.49  51.71  0  -29.33  2.60  0.03  -11.73  0 | 367.08  -11.66  -77.67  -8.99  .  -47.12  -22.74  -25.19  -37.28  . | 521.47  109.60  42.69  112.42  .  -11.54  27.94  25.26  13.81  . |

^a^ Reference group: no labelling, lower education; parameters estimates must be interpreted as difference with the reference group.

1. **Portion selection task questionnaire**

Items and answers to the portion selection task questionnaire are described **Table S10**.

**Table S10.** Description of the answers to the debriefing questionnaire overall and for participants of lower and higher education

|  | **All**  **(n=1,667)** | **Lower education**  **(n=810)** | **Higher education**  **(n=857)** | ***p*-value^a^** |
| --- | --- | --- | --- | --- |
| 1. The quantity I served myself for each dish was influenced by how many calories I thought were in the dishes | 3.1 ± 1.8 | 3.0 ± 1.7 | 3.3 ± 1.8 | < 0.001 |
| 2. The quantity I served myself for each dish was influenced by how much physical activity I thought I would have to do to burn off the calories that were I thought were in the dishes | 2.7 ± 1.6 | 2.6 ± 1.6 | 2.7 ± 1.7 | 0.101 |
| 3. On a typical day, knowing how many calories are in my meals would influence how much I eat | 3.3 ± 1.4 | 3.2 ± 1.4 | 3.4 ± 1.4 | 0.008 |
| 4. On a typical day, knowing how many calories are in my meals would influence how much physical activity I do | 2.9 ± 1.3 | 2.9 ± 1.3 | 2.9 ± 1.4 | 0.169 |
| 5. On a typical day, knowing how much physical activity I would have to do to burn off the calories that are in my meals would influence how much I eat | 3.0 ± 1.4 | 2.9 ± 1.5 | 3.0 ± 1.4 | 0.183 |
| 6. On a typical day, knowing how much physical activity I would have to do to burn off the calories that are in my meals would influence how much physical activity I do | 2.9 ± 1.4 | 2.9 ± 1.4 | 3.0 ± 1.4 | 0.425 |

We also performed two ANOVAs to test the effect of the labelling intervention and of highest educational qualification on the reported influence on self-served food quantity of the number of calories (item 1) and of the amount of physical activity needed to burn off the calories (item 2) (**Table S11**). Participants reported that their choices were influenced by the calories to a larger extent in all the labelling conditions compared to the no labelling condition. They also declared that their choices were influenced by physical activity to a larger extent in the PACE labelling and kcal and PACE labelling conditions compared to the no labelling condition.

**Table S11.** ANOVA models, dependant variables: items 1 and 2 of the portion selection task questionnaire (n=1667)

|  | **Type III effects** | | ***Estimate*** | ***95% LCL*** | ***95% UCL*** |
| --- | --- | --- | --- | --- | --- |
|  | ***F*** | ***p*** |  |  |  |
| **Item 1**  Intercept^a^  Intervention  Kcal labelling  PACE labelling  Kcal & PACE labelling  *No labelling*  Education  Higher  *Lower* | 14.77  11.43 | < 0.001  < 0.001 | 2.59  0.77  0.32  0.50  0  0.29  0 | 2.40  0.54  0.09  0.26  .  0.12  . | 2.77  1.00  0.55  0.73  .  0.45  . |
| **Item 2**  Intercept^a^  Intervention  Kcal labelling  PACE labelling  Kcal & PACE labelling  *No labelling*  Education  Higher  *Lower* | 24.84  3.46 | < 0.001  0.063 | 2.28  -0.003  0.79  0.38  0  0.14  0 | 2.11  -0.22  0.58  0.16  .  -0.008  . | 2.45  0.21  1.00  0.60  .  0.30  . |

^a^ Reference group: no labelling, lower education; parameters estimates must be interpreted as difference with the reference group.
